# Long Non-coding RNA T-uc.189 Modulates Neural Progenitor Cell Fate by Regulating *Srsf3* During Mouse Cerebral Cortex Development

**DOI:** 10.3389/fnins.2021.709684

**Published:** 2021-07-20

**Authors:** Meng Zhang, Junjie Zhou, Li Jiao, Longjiang Xu, Lin Hou, Bin Yin, Boqin Qiang, Shuaiyao Lu, Pengcheng Shu, Xiaozhong Peng

**Affiliations:** ^1^Institute of Medical Biology, Chinese Academy of Medical Sciences, and Peking Union Medical College, Kunming, China; ^2^The State Key Laboratory of Medical Molecular Biology, Department of Molecular Biology and Biochemistry, Institute of Basic Medical Sciences, Chinese Academy of Medical Sciences and Peking Union Medical College, Beijing, China

**Keywords:** neurogenesis, T-UCRs, T-uc.189, *Srsf3*, cortical development

## Abstract

Neurogenesis is a complex process that depends on the delicate regulation of spatial and temporal gene expression. In our previous study, we found that transcribed ultra-conserved regions (T-UCRs), a class of long non-coding RNAs that contain UCRs, are expressed in the developing nervous systems of mice, rhesus monkeys, and humans. In this study, we first detected the full-length sequence of T-uc.189, revealing that it was mainly concentrated in the ventricular zone (VZ) and that its expression decreased as the brain matured. Moreover, we demonstrated that knockdown of T-uc.189 inhibited neurogenesis. In addition, we found that T-uc.189 positively regulated the expression of serine-arginine-rich splicing factor 3 (*Srsf3*). Taken together, our results are the first to demonstrate that T-uc.189 regulates the expression of *Srsf3* to maintain normal neurogenesis during cortical development.

## Introduction

The mammalian neocortex is an evolved and complicated structure that is responsible for higher-order and complex brain physiological functions, such as cognition, sensory function, attention, memory, emotion, language, learning, motor function and perception. Mammalian neocortex formation, a complex and highly regulated developmental process, involves precise control of the differentiation and proliferation of neural progenitor cells (NPCs) (Kriegstein and Noctor, 2004; Evsyukova et al., 2013). The developing neocortex in the embryonic mouse brain contains the ventricular zone (VZ), subventricular zone (SV), intermediate zone (IZ) and cortical plate (CP). During the development of the cerebral cortical, neural stem cells transform from neuroepithelial (NE) cells to radial glial cells (RGCs), which are mainly located in the VZ of the forebrain. RGCs can divide in a symmetric way to amplify the progenitor pool, and can also generate one neuron and one RGC by asymmetric divisions. The orchestrated migration of newly generated neurons is the basis for the correct cortical hierarchy. The laminar structure of the neocortex develops via an inside-out construction pattern of migration, with deeper layers developing first and superficial layers developing last (Ayala et al., 2007; He et al., 2015). The proliferation and differentiation of NPCs and the migration of neuronal cells are fundamental biological processes that underlie normal brain formation and function, and some neurological diseases and brain injuries are caused by aberrant cerebral cortex development (Valiente and Marín, 2010; Liu, 2011; Reiner et al., 2016; Buchsbaum and Cappello, 2019; Francis and Cappello, 2020); however, the exact molecular mechanisms underlying this complex process are not yet well understood. Brain functions have become more complicated with species evolution, as the proportion of protein-coding genes has decreased significantly and the proportion of non-coding RNAs (ncRNAs) has increased significantly (Guttman et al., 2009). Understanding the roles of ncRNAs in the structural and functional development of the brain will be helpful to understand brain development processes and uncover metabolic diseases associated with brain development abnormalities. Moreover, ncRNAs are a class of RNAs that are not normally translated into proteins and include microRNAs, circular RNAs, small interfering RNAs (siRNAs), and long non-coding RNAs (lncRNAs) (Anastasiadou et al., 2018). LncRNAs refer to ncRNAs greater than 200 nucleotides in length and can regulate gene expression at multiple levels, such as at the transcriptional, posttranscriptional and epigenetic levels (Qian et al., 2019). Some brain-specific lncRNAs tend to have better sequence and transcriptional conservation, which is speculated to play a significant role in the structural and functional development of the brain (van Leeuwen and Mikkers, 2010; Wu et al., 2013; Francescatto et al., 2014; He et al., 2014; Washietl et al., 2014; Andersen et al., 2019; McDonel and Guttman, 2019).

Ultra-conserved elements (UCEs) are a remarkable class of DNA elements that were first discovered by Bejerano et al. (2004), who revealed that 481 genomic segments of at least 200 nt in length are absolutely conserved across the human, rat, and mouse genomes. In normal mammalian tissues and organs, approximately 93% of UCRs can be identified by their transcriptional activity, and these transcripts are termed transcribed UCRs (T-UCRs) (Calin et al., 2007). According to previous reports, T-UCRs expression and function are associated with the maintenance of normal life activities and the occurrence and development of various malignant tumors (Scaruffi et al., 2009; Mestdagh et al., 2010; Ferdin et al., 2013; Peng et al., 2013; Galasso et al., 2014; Jiang et al., 2016; Wu et al., 2016). Furthermore, some intragenic T-UCRs are correlated with their host genes and may regulate their splicing and transcription (Mehta et al., 2014). However, studies on the roles of T-UCRs in mammalian nervous system development and the maintenance of normal physiological functions are less informative. In our previous work, we have performed RT-PCR experiment to detect the expression signals of 76 T-UCRs in mice, rhesus monkeys and humans and found that 66% of them simultaneous expression among three species. Then we identified the spatiotemporal expression patterns of 50 T-UCRs during mouse brain development used ISH experimentation, revealing that 30% showed dynamic and relatively high expression. In this study, we thoroughly investigated the role of a representative UCR, T-uc.189. We aimed to systematically and thoroughly study its temporal and spatial expression profile characteristics and biological functions during mouse cerebral cortex development. This T-UCR was selected because of its sequence and expression conservation in the nervous systems of the abovementioned species, and its UCR sequence is located in the intronic region of the protein-coding gene Srsf3. *Srsf3* is an extremely important RNA splicing factor that helps to regulate the expression of many important genes and plays a role in the occurrence and development of a variety of cancers (Kumar et al., 2019; Fuentes-Fayos et al., 2020; Zhang et al., 2021). The latest study identified *Srsf3* as a potential cancer treatment target (Zhou et al., 2020). In this study, we first identified the full-length sequences of T-uc.189 by 3′- and 5′-RACE and northern blot analyses and then mapped the spatial and temporal expression profiles of T-uc.189 and its host gene *Srsf3* during the early stage of embryonic brain development. Moreover, knockdown of T-uc.189 *in vivo* suppressed neurogenesis, and T-uc.189 positively regulated the mRNA and protein expression of *Srsf3*. In addition, knockdown of *Srsf3* promoted NPCs proliferation, while overexpression of *Srsf3* restored the effect of T-uc.189 knockdown. These experimental results indicate that T-uc.189 could positively regulate the expression of *Srsf3* and play an important biological role in the development of the mouse cerebral cortex.

## Materials and Methods

### Animals

ICR mice were used for the *in vivo* experiment and were maintained at the Animal Centre of the Institute of Medical Biology, Chinese Academy of Medical Sciences in Kunming. The animal care and experiment were approved by the Institutional Animal Care and Use Committee of the Chinese Academy of Medical Sciences and performed in accordance with the institutional guidelines of the Chinese Academy of Medical Sciences and Peking Union Medical College.

### RACE Analysis

The 5′- and 3′-RACE experiments were performed using the SMARTer^®^, RACE 5′/3′ Kit (Takara, CA, United States) according to the manufacturer’s instructions. The RACE PCR products were separated on a 1.2% agarose gel and then cloned into pMD19-T vectors and sequenced. The gene-specific RACE primers are listed in Supplementary Table 2.

### Northern Blot Analysis

RNA was extracted from neural stem cells using the Poly(A) Tract mRNA Isolation Kit (Invitrogen, Carlsbad, CA, United States). Northern blot experiments were conducted using a digoxigenin (DIG) Northern Starter Kit (Roche, Basel, Switzerland) according to the manufacturer’s instructions.

### *In situ* Hybridization (ISH)

Timed pregnant mouse embryonic brains were fixed with 4% paraformaldehyde (PFA) in phosphate buffered saline (PBS). Then, the fixed tissues were cryoprotected with 25% sucrose in PBS and equilibrated in the O.C.T. Compound. Cryosections were cut at a thickness of 16 microns on a Leica CM1950 cryostat (Germany) and stored at −80°C. Then, the cryosections were incubated with DIG-labeled RNA probes and developed as previously described (Zhou et al., 2017). The primers are listed in Supplementary Table 2.

### Nuclear and Cytoplasmic Separation

Nuclear and cytoplasmic extraction reagents (NE-PER, Thermo Scientific, Waltham, MA, United States) were used to separate the nucleus and cytoplasm, and RNA was extracted from NIE-115 cells.

### RNA Fluorescence *in situ* Hybridization (FISH)

This assay was performed in a similar manner to the ISH assay described above using anti-digoxigenin-POD (Roche, Basel, Switzerland) and the TSA Plus Fluorescein System (Perkin Elmer, Waltham, MA, United States).

### *In utero* Electroporation

For *in vivo* transfection experiments, T-uc.189 and *Srsf3* knockdown constructs were generated with the pll3.7 vector, and *Srsf3* overexpression constructs were generated in the pCIG vector provided by Weimin Zhong (Yale University). The shRNA sequence is listed in Supplementary Table 2. All plasmids were extracted using the Endofree plasmid Maxi Kit (QIAGEN, Duesseldorf, Germany) and then spiked with Fast Green (Sigma-Aldrich, St. Louis, MO, United States). Pregnant dams (E13.5) were anesthetized by 4% isoflurane, and the anaesthetization was maintained by 2% isoflurane throughout the procedure. Cells were electroporated with electric pulses of 20–30 V for 50 ms five times at 950 ms intervals using the CUY21EDIT II electroporator.

### Immunohistochemistry

Immunohistochemistry (IHC) analyses of the fetal brain cryosections were performed as previously described (Shu et al., 2017). Nuclei were stained with DAPI (Sigma-Aldrich, St. Louis, MO, United States) and mounted with a coverslip. Pax6 (Convance, Princeton, NJ, United States), Tbr2 (Abcam, Cambridge, MA, United States) and NeuroD2 (Invitrogen, Carlsbad, CA, United States) were used as the IHC primary antibodies, while Alexa Fluor 594 (Invitrogen, Carlsbad, CA, United States) served as the secondary antibody. EdU staining was performed using a Click-iT^TM^ EdU 647 Imaging Kit (Invitrogen, Carlsbad, CA, United States) according to the manufacturer’s protocols. Images were collected using a Leica TCS SP8 microscope (Germany), and three independent experiments were performed for each group.

### Western Blot Analysis

Total protein was extracted from cultured cells with protein lysis buffer (50 mmol/L Tris, pH 7.5, 150 mmol/L NaCl, 2 mmol/L EDTA and 1% Triton X-100) supplemented with protease inhibitors (Roche, Basel, Switzerland). Proteins were separated by sodium dodecyl sulfate polyacrylamide gel electrophoresis (SDS-PAGE) and then subjected to Western blot analysis using a *Srsf3* antibody (Invitrogen, Carlsbad, CA, United States) and a Tubulin antibody (GeneTex, San Antonio, Texas, United States). Three independent experiments were performed for each group.

### Cell Culture and Transfection

N1E-115 cells were provided by Dr. Yan Zhou (Wuhan University) and cultured at 37°C and 5% CO_2_ in Dulbecco’s modified Eagle’s medium (DMEM) (Thermo Scientific, Waltham, MA, United States) supplemented with 10% (v/v) foetal bovine serum (FBS) (Gibco, Grand Island, NY, United States). To construct the overexpression and knockdown cell lines, plasmids were transfected using Lipofectamine 3000 (Invitrogen, Carlsbad, CA, United States) according to the manufacturer’s instructions.

### RNA Isolation and Quantitative Real-Time PCR (qRT-PCR) Analysis

For qRT-PCR analysis, total RNA was extracted using TRIzol reagent (Invitrogen, Carlsbad, CA, United States) and quantitated with NANODROP ONE. First-strand cDNA synthesis was performed using a reverse transcriptase cDNA synthesis kit (Takara, Tokyo, Japan) according to the manufacturer’s instructions. Real-time PCR was carried out using Supermix (Bio-Rad, Hercules, CA, United States) according to the manufacturer’s protocols. To ensure the reliability and credibility of the results, the experiments were repeated at least three times independently. The mouse GAPDH gene was amplified as a control. The primer sequences are listed in Supplementary Table 2. Three independent experiments were performed for each group.

### Statistical Analysis

The results are expressed as the mean ± standard deviation. Statistical analyses were performed using GraphPad Prism 8, and Student’s *t*-test was used for comparison between the two groups, and ANOVA was used for the comparison between multiple groups. *P*-values <0.05 were considered statistically significant.

## Results

### T-uc.189 Is Mainly Expressed in the Ventricular Zone During Neocortical Development and Primarily Localized in the Nucleus

**FIGURE 1:**
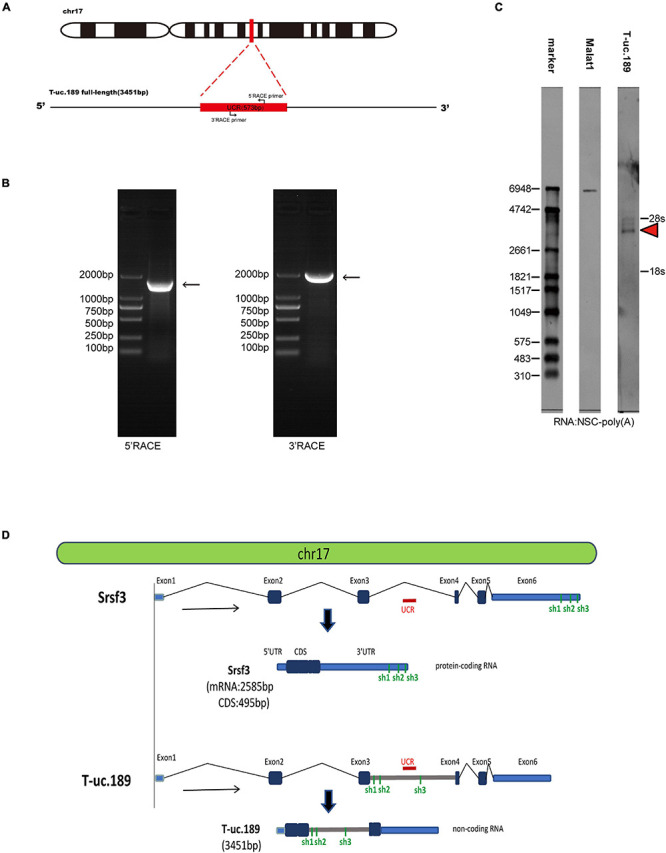
Information regarding the full-length sequence of T-uc.189. **(A)** Schematic diagrams of the RACE assays. **(B)** Agarose gel electrophoresis image of the nested PCR products from the 5′- and 3′-RACE assays. **(C)** Northern blot experiments with total RNA extracted from neural stem cells. The red arrow indicates the RNA signals of T-uc.189. **(D)** The genomic location of T-uc.189 relative to that of its host gene *Srsf3*. The red line indicates the position of the ultra-conserved region of T-uc.189. The green line indicates the position of the shRNA of *Srsf3* and T-uc.189.

To investigate the T-uc.189 profile during mouse cerebral cortex development, we first detected the genomic location of T-uc.189, revealing that its UCR is located in the intronic region of the *Srsf3* gene. Then, 3′- and 5′-RACE and Northern blot experiments were used to identify the 3451 bp full-length sequence of T-uc.189 (Figures 1A–C and Supplementary Figures 1A,B). We compared the genomic and sequence locations of the *Srsf3* gene and T-uc.189 (Figure 1D). T-uc.189 was not identical to the ten published *Srsf3* transcript sequences (Supplementary Figure 2A). Then, the transcript in fetal mouse brain cDNA was detected by PCR and Sanger sequencing (Supplementary Figures 3A–C). Moreover, the protein-coding ability of T-uc.189 was evaluated using the Coding-Potential Assessment Tool (CPAT) and the Coding Potential Calculator (CPC), revealing a weak protein-coding potential (Supplementary Table 1). T-uc.189 contained one open reading frame (ORF) encoding a putative protein of 129 amino acids that was identical to the predicted ORF of *srsf3*-204 (ncRNA). The same two intron-retained transcripts were not expected to encode a protein due to the C-terminal truncations compared with *srsf3*-202 (a protein-coding gene) (Supplementary Figure 2B). These data provide evidence that T-uc.189 constitutes a novel lncRNA that may serve a regulatory function.

**FIGURE 2:**
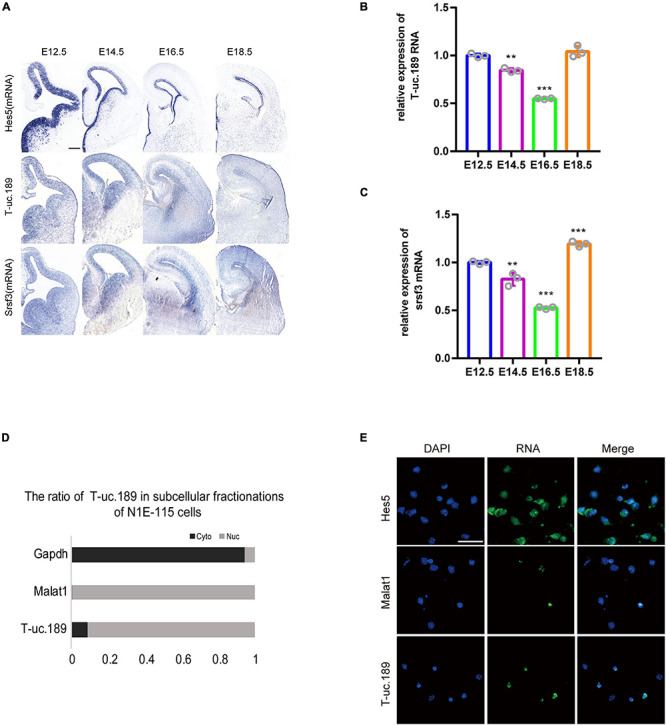
Expression pattern of T-uc.189. **(A)** In mouse fetal brains at E12.5, E14.5, E16.5 and E18.5, the spatiotemporal expression patterns of T-uc.189 and *Srsf3* were determined by RNA *in situ* hybridization, and Hes5 served as a positive control. The scale bar is 200 μm. **(B,C)** The relative expression of T-uc.189 and Srsf3 were detected by real-time PCR on fetal mouse brains at different stages (E12.5, E14.5, E16.5, E18.5). **(D)** Nucleocytoplasmic separation experiments were performed in NIE-115 cells to determine the distribution of T-uc.189. **(E)** Combined immunofluorescence/RNA fluorescence *in situ* hybridization (IF/RNA FISH) was performed to detect the subcellular localization of T-uc.189 in neural stem cells. The scale bar is 30 μm. *n* ≥ 3 independent biological repeats. The results are expressed as the mean ± SD, and comparisons were performed by ANOVA. The statistically significant *P* values are shown as **P < 0.01 or ***P < 0.001.

To investigate the spatiotemporal expression profiles of T-uc.189 and *Srsf3*, we performed ISH experiments with locked nucleic acid (LNA)-modified probes and real-time PCR on fetal mouse brains at different stages (E12.5, E14.5, E16.5, E18.5). T-uc.189 and *Srsf3* were expressed at relatively high levels in the cerebral cortex VZs, and their mRNA expression gradually decreased as embryonic stage 12.5 days to 16.5 days (Figures 2A–C). N1E-115 cells were subjected to cytoplasmic and nuclear fractionation, and RNA FISH analysis of T-uc.189 from neural stem cells showed that T-uc.189 was mainly expressed in the nucleus (Figures 2D,E). Given the results observed above, we hypothesized that T-uc.189 is involved in cerebral cortex development via transcriptional regulation.

### Knockdown of T-uc.189 Perturbs Neurogenesis

**FIGURE 3:**
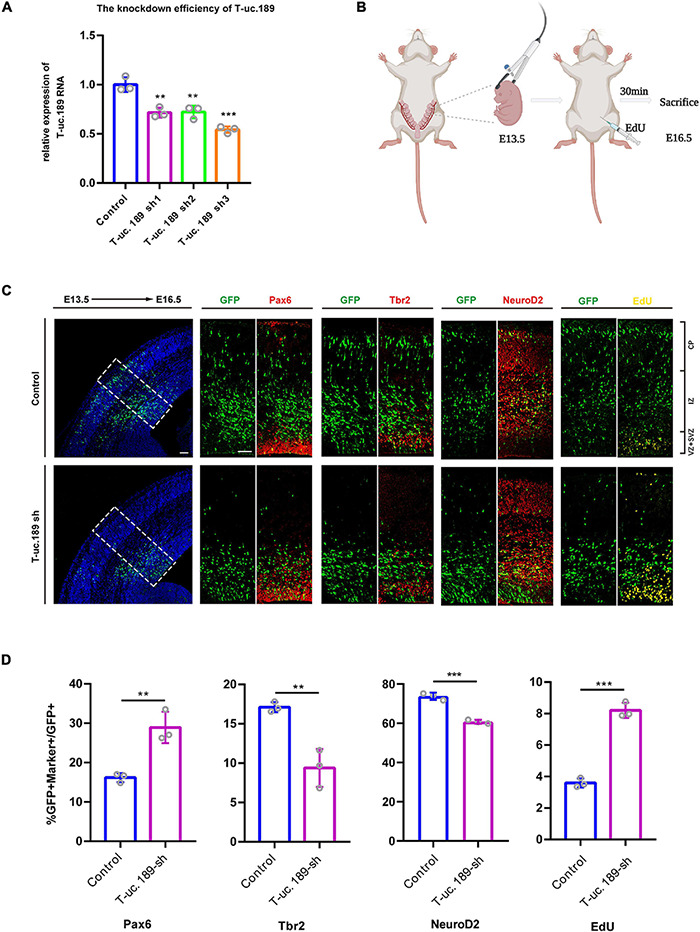
Knockdown of T-uc.189 inhibits neurogenesis. **(A)** The knockdown efficiency of shRNA targeting T-uc.189 was confirmed by real-time PCR analysis. **(B)** Schematic diagrams of the *in utero* electroporation assay created with BioRender.com. **(C)** T-uc.189 knockdown and control plasmids were electroporated at E13.5 forebrains, and brain sections at E16.5 were stained with Pax6, Tbr2, and NeuroD2 antibodies or with an EdU staining kit. The dashed line in the left panel indicates the region in which transfected cells were distributed. CP, cortical plate; IZ, intermediate zone; SVZ, subventricular zone; VZ, ventricular zone. The scale bar is 75 μm. **(D)** Quantification of GFP^+^ cells co-expressing the markers. *n* ≥ 3 independent biological repeats. The results are expressed as the mean ± SD, and comparisons were performed by Student’s *t*-test or ANOVA. The statistically significant *P* values are shown as **P < 0.01 or ***P < 0.001.

To gain insight into the function of T-uc.189 during neocortical development, we first constructed a T-uc.189 knockdown plasmid with the pLL3.7 vector and performed transfection and real-time PCR to assess the knockdown efficiency in N1E-115 cells. We found that shRNA3 exhibited the highest interference effect and was therefore selected for the following experiments (Figure 3A).

To better understand the possible role of T-uc.189 during mouse neocortical development, *in utero* electroporation (IUE) was performed to introduce control and T-uc.189 knockdown plasmids into proliferating cells in the dorsal forebrain region at E13.5. Embryonic brains were collected after 72 h at E16.5 (Figure 3B), and electroporated cells expressing GFP were identified by tissue immunofluorescence analysis. To determine whether T-uc.189 can determine the fate of NPCs, we examined GFP^+^ cells by co-staining with Pax6 (a RGC marker), Tbr2 (an intermediate neural progenitor (INP) marker) and NeuroD2 (a neuronal marker) and found an increased percentage of Pax6^+^GFP^+^/GFP^+^ cells but significantly decreased percentages of Tbr2^+^GFP^+^/GFP^+^ and NeuroD2^+^GFP^+^/GFP^+^ cells compared with the control group (Figures 3C,D). Thus, these results indicate that knockdown of T-uc.189 suppressed the differentiation of NPCs into neurons. To explore the role of T-uc.189 in NPCs proliferation, electroporated pregnant mice were intraperitoneally injected with 5-ethynyl-2′-deoxyuridine (EdU) at 30 min prior to being sacrificed (Figure 3B). Knockdown of T-uc.189 increased the proportion of GFP^+^EdU^+^ cells compared to that in the control group (Figures 3C,D). These results show that knockdown of T-uc.189 promotes the proliferation of NPCs. Similar results were obtained using shRNA2 of T-uc.189 (Supplementary Figure 5), excluding the possibility of off-target effects.

### T-uc.189 Positively Regulates the Expression of *Srsf3*

Intragenic T-UCRs are generally considered to regulate the expression of their host genes. Therefore, to assess the regulatory relationship between T-uc.189 and *Srsf3*, we constructed one T-uc.189 overexpression plasmid and three T-uc.189 knockdown plasmids and detected their efficiencies by real-time PCR (Figure 3A and Supplementary Figure 4A). Next, we examined whether the levels of *Srsf3* mRNA and protein were correlated with T-uc.189 expression in N1E-115 cells.

**FIGURE 4:**
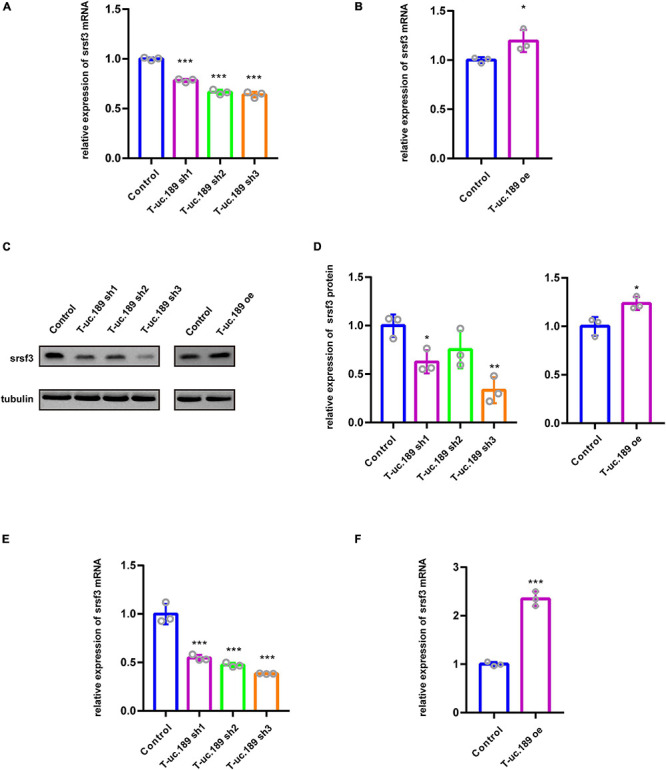
T-uc.189 positively regulates the expression of *Srsf3*. **(A)** The relative mRNA expression of *Srsf3* in T-uc.189 knockdown NIE-115 cells compared with the control. **(B)** The relative mRNA expression of *Srsf3* in T-uc.189-overexpressing NIE-115 cells compared with the control. **(C)** Western blot assay of the relative protein expression of *Srsf3* in T-uc.189 knockdown and overexpression NIE-115 cells compared with the control. **(D)** Statistical analysis of *Srsf3* protein expression as determined by Western blot. **(E)** The relative mRNA expression of Srsf3 of T-uc.189 knockdown in the cortex compared with the control. **(F)** The relative mRNA expression of Srsf3 of T-uc.189-overexpression in the cortex compared with the control. *n* ≥ 3 independent biological repeats. The results are expressed as the mean ± SD, and comparisons were performed by Student’s *t*-test or ANOVA. The statistically significant *P* values are shown as *P < 0.05, **P < 0.01 or ***P < 0.001.

We found that the mRNA expression level of *Srsf3* was significantly decreased in the cells treated with T-uc.189 knockdown plasmids compared with the cells treated with the control plasmids (Figure 4A). Compared with that in control cells, the mRNA expression level of *Srsf3* was significantly increased in N1E-115 cells transfected with the T-uc.189 overexpression plasmid (Figure 4B). Next, Western blot experiments were performed to assess the regulatory effect of T-uc.189 on protein expression level of *Srsf3*. In line with the regulation at the RNA level, inhibition of T-uc.189 downregulated *Srsf3* protein expression, while overexpression of T-uc.189 upregulated Srsf3 protein expression (Figures 4C,D). Furthermore, *Srsf3* knockdown and overexpression plasmids were constructed and transfected into N1E-115 cells, and their efficiencies were detected by real-time PCR (Supplementary Figures 4B,C). In contrast, the expression of T-uc.189 was not affected by *Srsf3* (Supplementary Figures 4D,E). To further verify the regulation relationship between T-uc.189 and Srsf3, we conducted IUE followed by real-time PCR. The mRNA expression level of Srsf3 is positively regulated by T-uc.189 in the cortex, which is consistent with the *in vitro* data (Figures 4E,F and Supplementary Figures 4F–I). Taken together, our results indicate that T-uc.189 positively regulates the expression of *Srsf3* and likely exerts its biological functions upstream of *Srsf3*.

### Knockdown of *Srsf3* Promotes NPCs Proliferation

**FIGURE 5:**
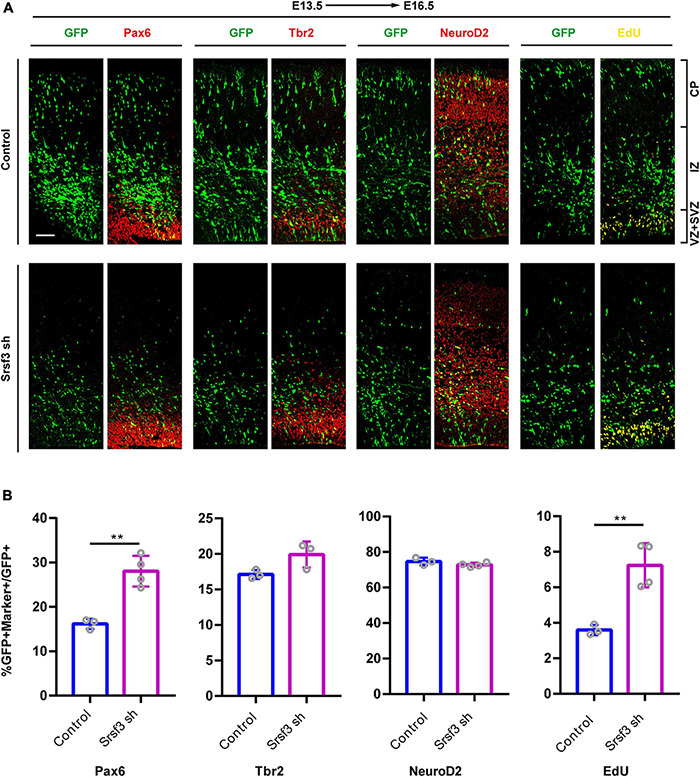
Knockdown of *Srsf3* promotes NPCs proliferation. **(A)** *Srsf3* knockdown and control plasmids were electroporated into mouse forebrains at E13.5, and brain sections at E16.5 were stained with Pax6, Tbr2, and NeuroD2 antibodies or with an EdU staining kit. CP, cortical plate; IZ, intermediate zone; SVZ, subventricular zone; VZ, ventricular zone. The scale bar is 75 μm. **(B)** Quantification of GFP^+^ cells co-expressing the markers. *n* ≥ 3 independent biological repeats. The results are expressed as the mean ± SD, and comparisons were performed by Student’s *t*-test. The statistically significant *P* values are shown as **P < 0.01.

To further confirm whether the loss of *Srsf3* expression also disrupts cortical development, we conducted IUE experiments at E13.5 using GFP-expressing shRNA1 plasmids targeting *Srsf3*. These cells were followed with the RGC marker Pax6, the INP marker Tbr2, the neuronal marker NeuroD2 and the proliferation marker EdU (Figure 5A). The co-staining of GFP^+^ cells with Pax6, Tbr2, NeuroD2 and EdU revealed that *Srsf3* knockdown increased the proportions of GFP^+^Pax6^+^/GFP^+^ and GFP^+^EdU^+^/GFP^+^ cells but did not affect the proportions of Tbr2^+^GFP^+^/GFP^+^ and NeuroD2^+^GFP^+^/GFP^+^ cells compared with the control (Figure 5B). These results indicate that knockdown of *Srsf3* does not affect the differentiation of NPCs but does promote their proliferation. Similar results were obtained using shRNA2 of *Srsf3* (Supplementary Figure 5), excluding the possibility of off-target effects. Overall, these abnormal influences of *Srsf3* knockdown on neurogenesis were generally similar to those of T-uc.189.

### *Srsf3* Ameliorates the Perturbation of NPCs Proliferation Induced by T-uc.189 Deficiency

**FIGURE 6:**
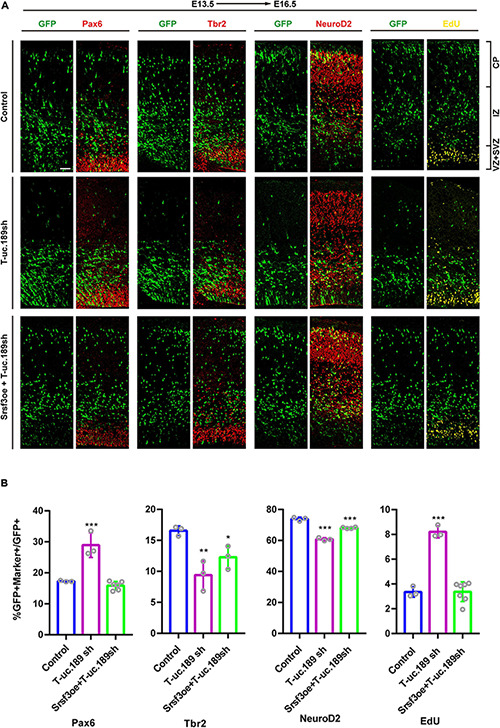
*Srsf3* overexpression ameliorates the knockdown effects of T-uc.189 on NPCs proliferation. **(A)** T-uc.189 knockdown plasmids together with *Srsf3* overexpression, T-uc.189 knockdown plasmids and control plasmids were electroporated into mouse forebrains at E13.5, and the mouse brain sections were stained with Pax6, Tbr2, and NeuroD2 antibodies or with an EdU staining kit at E16.5. CP, cortical plate; IZ, intermediate zone; SVZ, subventricular zone; VZ, ventricular zone. The scale bar is 50 μm. **(B)** Quantification of GFP^+^ cells co-expressing the markers. *n* ≥ 3 independent biological repeats. The results are expressed as the mean ± SD, and comparisons were performed by ANOVA. The statistically significant *P* values are shown as *P < 0.05, **P < 0.01 or ***P < 0.001.

To determine whether *Srsf3* is a downstream target of T-uc.189 during neurogenesis, we conducted *in vivo* rescue experiments to confirm the relationship between T-uc.189 and *Srsf3*. We co-electroporated T-uc.189 knockdown and *Srsf3* overexpression plasmids at E13.5 (Figure 6A). Compared with that of control cells, the proportions of EdU^+^GFP^+^/GFP^+^ and Pax6^+^GFP^+^/GFP^+^ cells were similar, while the proportions of Tbr2^+^GFP^+^/GFP^+^ and NeuroD2^+^GFP^+^/GFP^+^ cells were decreased (Figure 6B). These results indicate that *Srsf3* overexpression rescued the abnormal effect of T-uc.189 deficiency on NPCs proliferation.

## Discussion

A total of 481 segments longer than 200 base pairs have been identified as UCRs and are absolutely conserved in the mouse, rat and human genomes (Bejerano et al., 2004). The transcripts of UCRs are called T-UCRs, a novel class of lncRNAs that have characteristics differing from those of traditional ncRNAs (St Laurent et al., 2015). T-UCRs expression patterns are highly conserved across species and are specific to some tissues. T-UCRs may positively or negatively regulate the transcription of their host genes.

Previous reports have shown that many UCRs are expressed as enhancers in various regions of the nervous system development and T-UCRs play an important role in the pathogenesis of cancer (Pennacchio et al., 2006; Peng et al., 2013; Bo et al., 2016; Goto et al., 2016), and their functional importance in human development and disease is being increasingly recognized. In our previous study, we found that T-UCRs have high levels of not only sequence conservation but also expression conservation in the nervous system during the evolution of various species (Zhou et al., 2017). Previous studies have reported that many T-UCRs are expressed in the rat cerebral cortex and most likely play an essential role in physiological brain functions (Mehta et al., 2014). Previous study has shown that a T-UCR, T-uc.170, is expressed in the forebrain and could regulate the proliferation of NPCs during neurogenesis *in vivo* (Pascale et al., 2020).

In this study, we identified a novel lncRNA, T-uc.189, that influences neurogenesis. We identified the full-length sequence and detected the potential coding capacity of T-uc.189. T-uc.189 was shown to be expressed in the VZ during cortex development, and *in vivo* experiments showed that T-uc.189 knockdown promoted the self-renewal capacity of NPCs and inhibited their differentiation, indicating that T-uc.189 is essential for maintaining the balance between NPC proliferation and differentiation. We also identified *Srsf3* as a regulatory target of T-uc.189 during mouse cortex development.

*Srsf3* (also called SRp20), a protein coding gene, is the smallest member of the pre-mRNA splicing factor family and is involved in processes such as RNA splicing, protein translation (Bedard et al., 2007), termination of transcription (Cui et al., 2008), and insulin signaling (Saeki et al., 2005). Previous studies identified *Srsf3* as playing an important role in embryonic development, and *Srsf3*-null mutant embryos failed to develop into blastocysts and died at the morula stage [Jumaa et al., 1999, 9(16)]. *Srsf3* is also involved in many neurological disorders due to its regulatory role in the alternative splicing of important genes (Goedert and Spillantini, 2000; Yu et al., 2004; Watanuki et al., 2008; Wong et al., 2012). However, the biological function of *Srsf3* in human neocortical development, its upstream regulator, and the manner of regulation during neurogenesis have not been revealed.

Herein, we first elucidated the regulatory mechanisms underlying the association between T-uc.189 and *Srsf3* expression. We further found that knockdown of *Srsf3* promoted NPCs proliferation, while overexpression of *Srsf3* ameliorated the effects of T-uc.189 on NPCs proliferation. However, knockdown of T-uc.189 suppressed the differentiation of NPCs into neurons and knockdown of *Srsf3* did not affect the NPCs differentiation process. And Srsf3 overexpression mostly rescued the phenotype induced by T-uc189 knockdown. In other words, T-uc.189 probably can also interact with other partners to regulate the NPCs differentiation to neurons. Taken together, our results demonstrate that the lncRNA T-uc.189 probably regulates and controls the expression of its downstream target *Srsf3* to maintain normal function during cortical development. In addition, our data showed that knockdown T-uc.189 or Srsf3 disturbed the distribution pattern of GFP^+^ cells. More GFP^+^ cells being located to the VZ/SVZ and a reduction in GFP^+^ cells in the cortical plate. Meanwhile, the rescue experiment ameliorated the distribution pattern. It could just be due to the changes in the fate of NPCs, as our results showed, or T-uc.189 or Srsf3 may have a potential role in migration. In future work, we will conduct experiments to explore the migration effects on the premise of excluding the factors affecting the fate of NPCs. Our study is a great first step, but a substantial amount of follow-up work remains. First, the sample size of the study is relatively small. We expected to expand the sample size in our future work and our findings could be strengthened. Second, the molecular mechanisms of T-uc.189, its role in NPCs differentiation and the detailed mechanism by which it regulates *Srsf3* expression need to be further investigated. Third, the exact molecular mechanisms by which *Srsf3* exerts its biological effect *in vivo* during cortex development also remain unknown.

Herein, we first explored whether T-uc.189 and *Srsf3* are crucial regulators of cortex development, revealing that T-uc.189 positively regulates the expression of *Srsf3* and underlies aberrant NPC fate. We revealed a new and comprehensive regulatory relationship between T-UCRs and their host genes during mammalian neocortical development. Exploring the precise molecular mechanisms by which T-uc.189 and *Srsf3* function during nervous system development will be helpful for understanding the mechanisms of *Srsf3*-related diseases and potential developmental defects. We hope that our work will provide some ideas for associated disease therapies.

## Data Availability Statement

The raw data supporting the conclusions of this article will be made available by the authors, without undue reservation.

## Ethics Statement

The animal study was reviewed and approved by The Institutional Animal Care and Use Committee (IACUC) of the Chinese Academy of Medical Sciences and Peking Union Medical College.

## Author Contributions

All authors listed have made a substantial, direct and intellectual contribution to the work, and approved it for publication.

## Conflict of Interest

The authors declare that the research was conducted in the absence of any commercial or financial relationships that could be construed as a potential conflict of interest.

## Supplementary Material

Supplementary Figure 1 Cloning of the full-length mouse lncRNA T-uc.189 gene. **(A)** Sequencing the PCR products from the 5′- and 3′-RACE assays revealed the boundary between the universal anchor primer and the T-uc.189 sequences. **(B)** Nucleotide sequence of the full-length mouse lncRNA T-uc.189 gene.

Supplementary Figure 2 Nucleotide and amino acid sequences of the ORF prediction alignment of T-uc.189. **(A)** Alignment of the T-uc.189 full-length sequence with the ten transcripts of *Srsf3*. **(B)** The predicted amino acid sequences of T-uc.189 and *Srsf3*-204 (non-coding RNA) aligned with *Srsf3*-202 (protein-coding gene).

Supplementary Figure 3 T-uc.189 was detected by RT-PCR and Sanger sequencing. **(A)** Total RNA was harvested from the E14.5 mouse brain and purified by DNase I. Then, the RNA was reverse transcribed into cDNA and verified by PCR. **(B,C)** T-uc.189 as identified by PCR and sequencing.

Supplementary Figure 4 *Srsf3* did not regulate T-uc.189 expression. **(A)** The T-uc.189 overexpression efficiency in N1E-115 cells was examined by real-time PCR. **(B)** The knockdown efficiency of the shRNA targeting *Srsf3* in N1E-115 cells was confirmed by real-time PCR. **(C)** The overexpression efficiency of *Srsf3* in N1E-115 cells was detected by real-time PCR. **(D)** The relative expression of T-uc.189 in *Srsf3* knockdown NIE-115 cells compared with the control. **(E)** The relative expression of T-uc.189 in *Srsf3*-overexpressing NIE-115 cells compared with the control (*n* ≥ 3 independent biological repeats). **(F)** The knockdown efficiency of the shRNA targeting T-uc.189 and the overexpression efficiency of T-uc.189 in the cortex were confirmed by real-time PCR. **(G)** The knockdown efficiency of the shRNA targeting *Srsf3* and the overexpression efficiency of Srsf3 in the cortex were confirmed by real-time PCR. **(H)** The relative expression of T-uc.189 of Srsf3 knockdown in the cortex compared with the control. **(I)** The relative expression of T-uc.189 of Srsf3-overexpression in the cortex compared with the control. *n* ≥ 3 independent biological repeats. The results are expressed as the mean ± SD, and comparisons were performed by Student’s *t*-test or ANOVA. The statistically significant *P* values are shown as ^∗^*P* < 0.05, ^∗∗^*P* < 0.01 or ^∗∗∗^*P* < 0.001.

Supplementary Figure 5 Knockdown of T-uc.189 or *Srsf3* inhibits neurogenesis. **(A)** T-uc.189 knockdown plasmids, *Srsf3* knockdown plasmids and control plasmids were electroporated into mouse forebrains at E13.5, and the mouse brain sections were stained with Pax6, Tbr2, and NeuroD2 antibodies or with an EdU staining kit at E16.5. CP, cortical plate; IZ, intermediate zone; SVZ, subventricular zone; VZ, ventricular zone. The scale bar is 50 μm. **(B)** Quantification of GFP^+^ cells co-expressing the markers. *n* ≥ 3 independent biological repeats. The results are expressed as the mean ± SD, and comparisons were performed by ANOVA. The statistically significant *P* values are shown as ^∗^*P* < 0.05, ^∗∗^*P* < 0.01 or ^∗∗∗^*P* < 0.001.
