# Supplementary figures and images for: Long Non-coding RNA T-uc.189 Modulates Neural Progenitor Cell Fate by Regulating Srsf3 During Mouse Cerebral Cortex Development
Source: Front Neurosci. 2021 Jul 20;15:709684. doi: 10.3389/fnins.2021.709684 (PMC8329457; doi:10.3389/fnins.2021.709684)

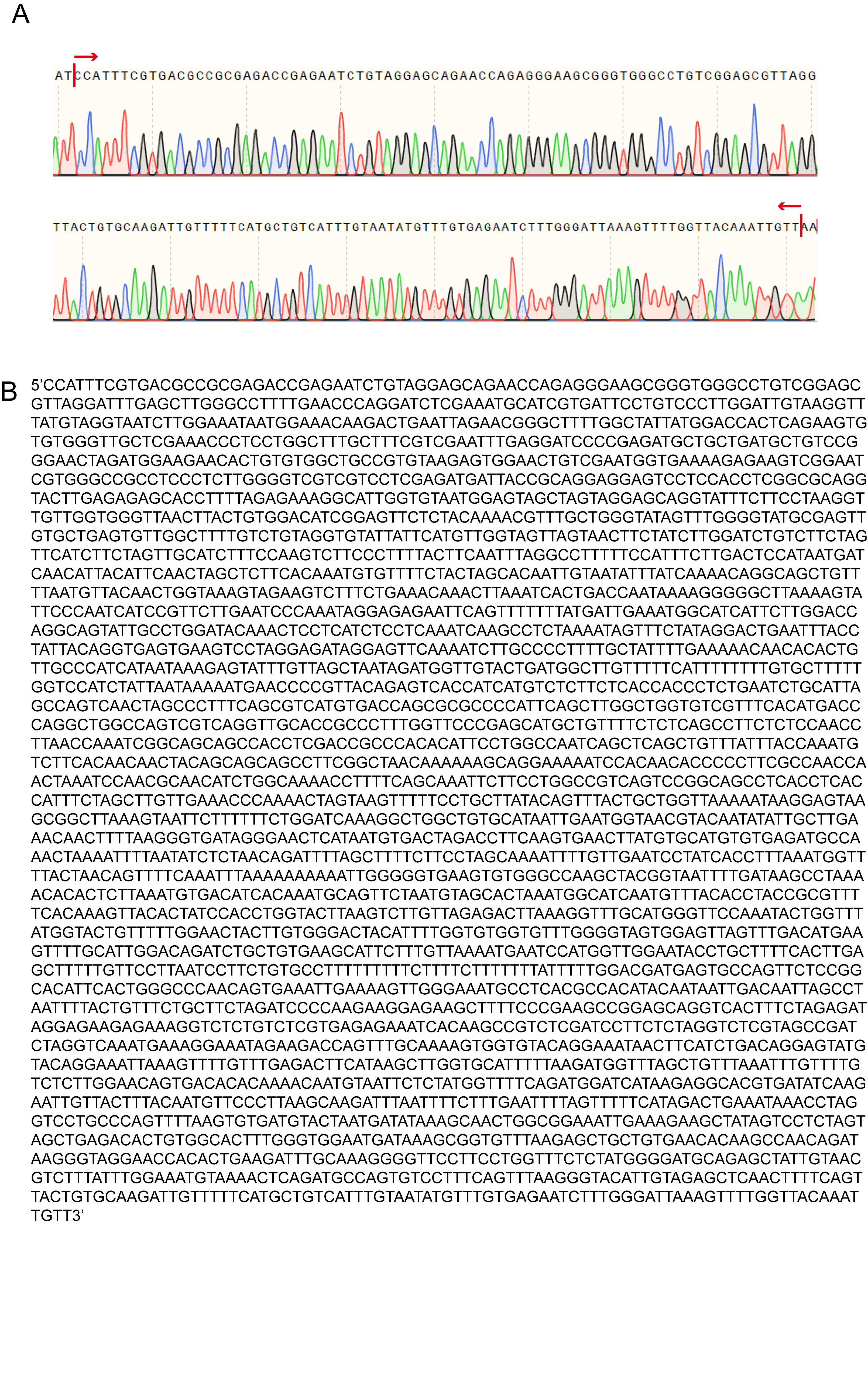

Supplement: Supplementary Figure 1 — Cloning of the full-length mouse lncRNA T-uc.189 gene. (A) Sequencing the PCR products from the 5′- and 3′-RACE assays revealed the boundary between the universal anchor primer and the T-uc.189 sequences. (B) Nucleotide sequence of the full-length mouse lncRNA T-uc.189 gene. [file Image_1.TIF]

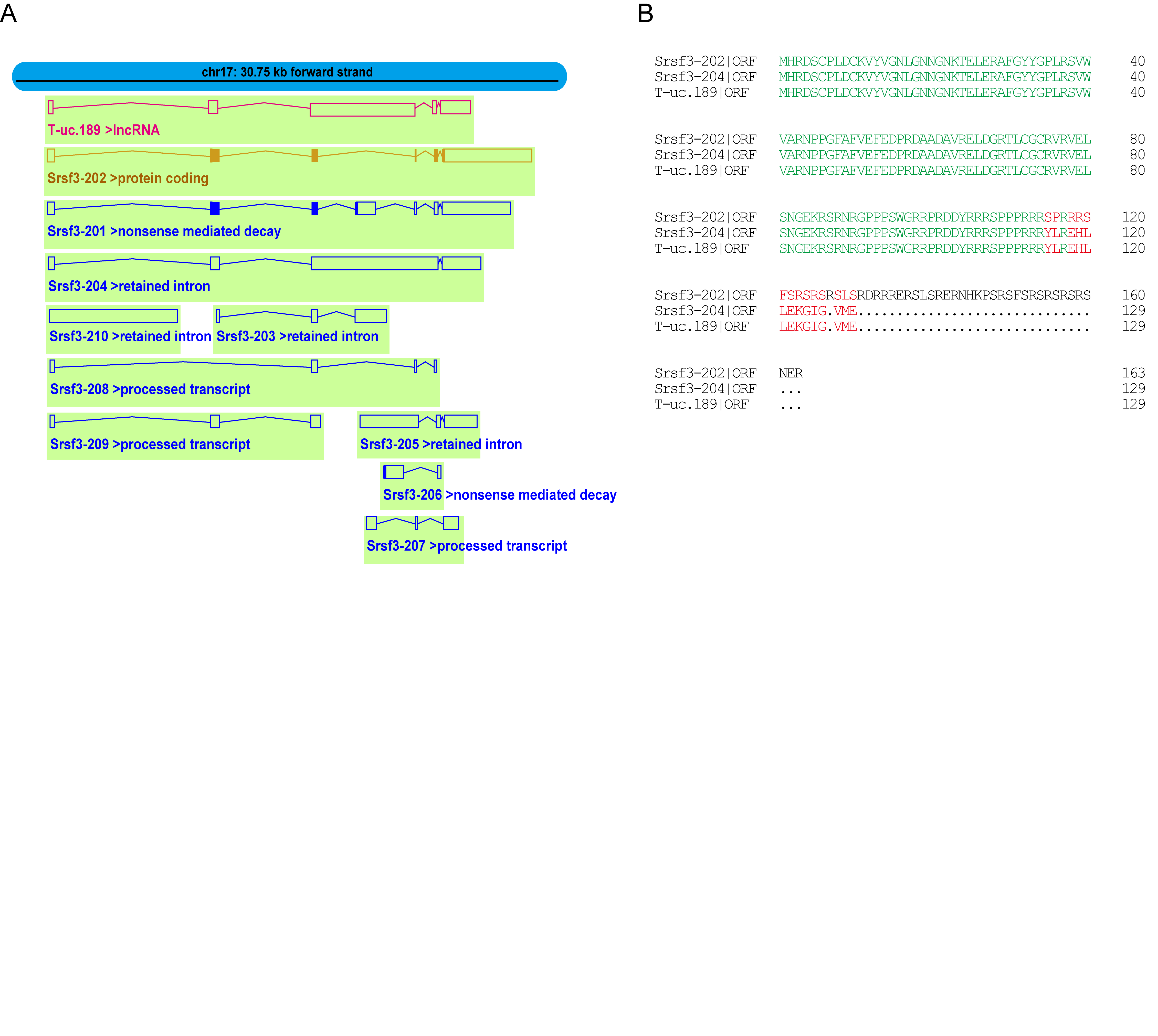

Supplement: Supplementary Figure 2 — Nucleotide and amino acid sequences of the ORF prediction alignment of T-uc.189. (A) Alignment of the T-uc.189 full-length sequence with the ten transcripts of Srsf3. (B) The predicted amino acid sequences of T-uc.189 and Srsf3-204 (non-coding RNA) aligned with Srsf3-202 (protein-coding gene). [file Image_2.TIF]

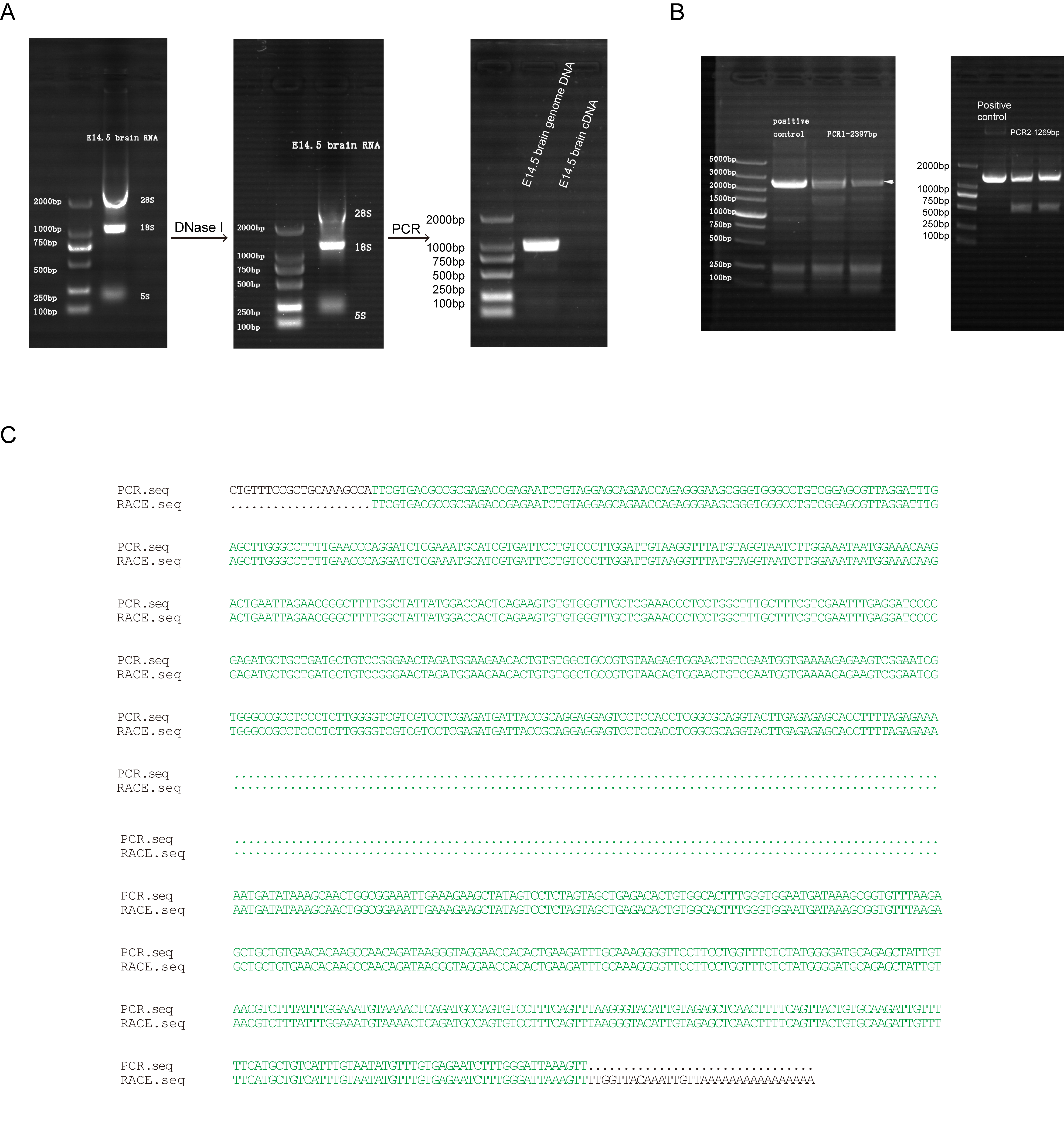

Supplement: Supplementary Figure 3 — T-uc.189 was detected by RT-PCR and Sanger sequencing. (A) Total RNA was harvested from the E14.5 mouse brain and purified by DNase I. Then, the RNA was reverse transcribed into cDNA and verified by PCR. (B,C) T-uc.189 as identified by PCR and sequencing. [file Image_3.TIF]

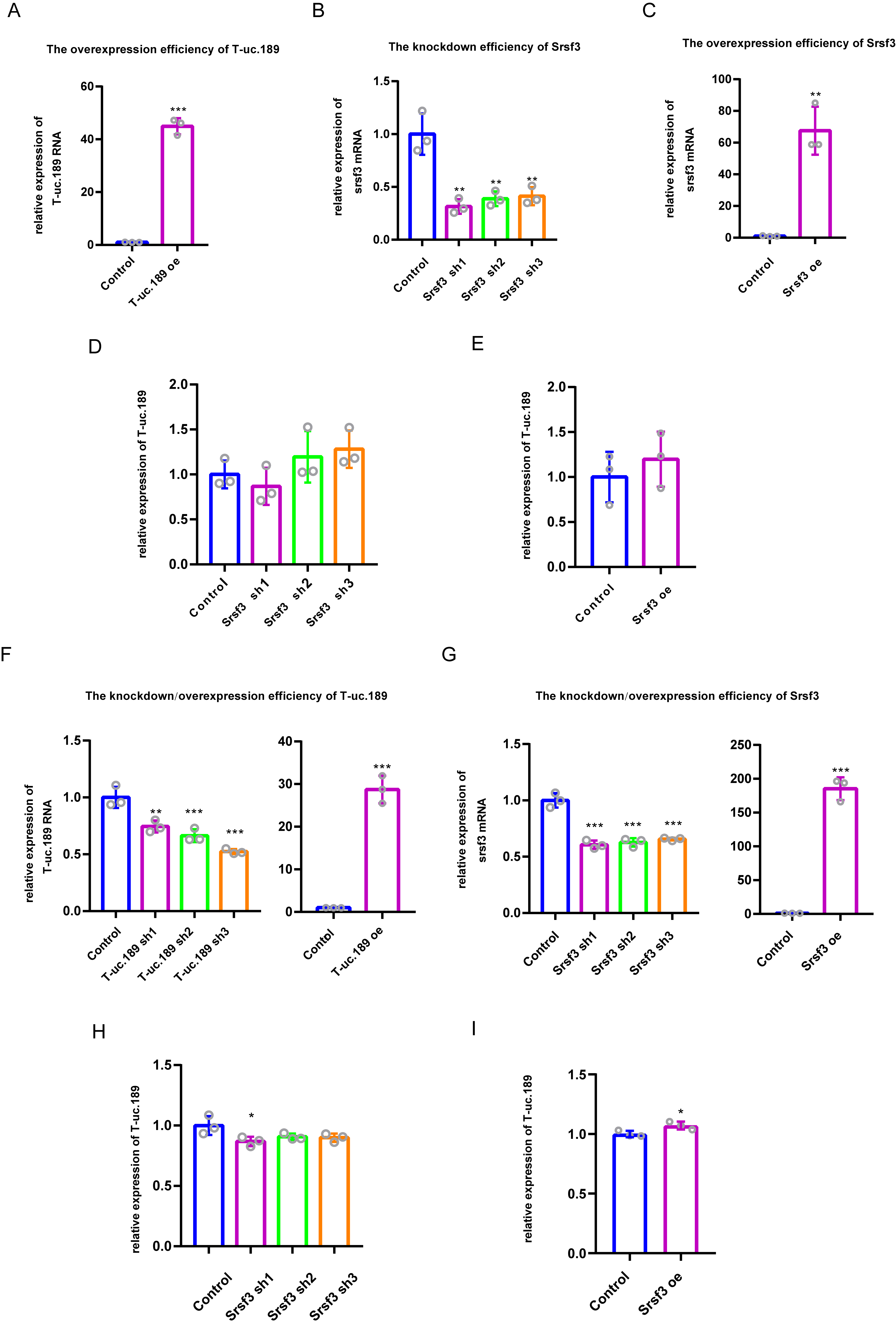

Supplement: Supplementary Figure 4 — Srsf3 did not regulate T-uc.189 expression. (A) The T-uc.189 overexpression efficiency in N1E-115 cells was examined by real-time PCR. (B) The knockdown efficiency of the shRNA targeting Srsf3 in N1E-115 cells was confirmed by real-time PCR. (C) The overexpression efficiency of Srsf3 in N1E-115 cells was detected by real-time PCR. (D) The relative expression of T-uc.189 in Srsf3 knockdown NIE-115 cells compared with the control. (E) The relative expression of T-uc.189 in Srsf3-overexpressing NIE-115 cells compared with the control (n ≥ 3 independent biological repeats). (F) The knockdown efficiency of the shRNA targeting T-uc.189 and the overexpression efficiency of T-uc.189 in the cortex were confirmed by real-time PCR. (G) The knockdown efficiency of the shRNA targeting Srsf3 and the overexpression efficiency of Srsf3 in the cortex were confirmed by real-time PCR. (H) The relative expression of T-uc.189 of Srsf3 knockdown in the cortex compared with the control. (I) The relative expression of T-uc.189 of Srsf3-overexpression in the cortex compared with the control. n ≥ 3 independent biological repeats. The results are expressed as the mean ± SD, and comparisons were performed by Student’s t-test or ANOVA. The statistically significant P values are shown as ∗P < 0.05, ∗∗P < 0.01 or ∗∗∗P < 0.001. [file Image_4.TIF]

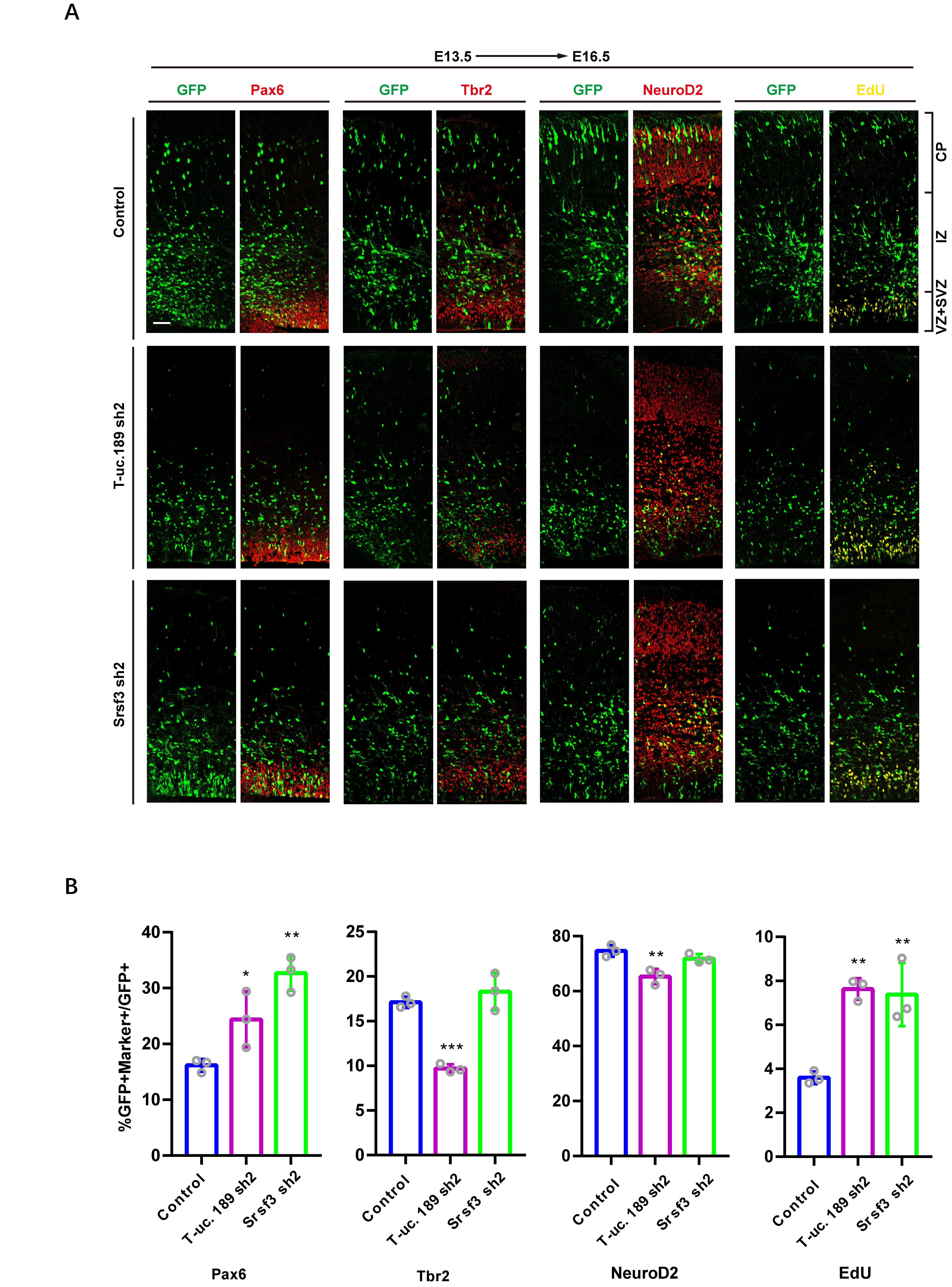

Supplement: Supplementary Figure 5 — Knockdown of T-uc.189 or Srsf3 inhibits neurogenesis. (A) T-uc.189 knockdown plasmids, Srsf3 knockdown plasmids and control plasmids were electroporated into mouse forebrains at E13.5, and the mouse brain sections were stained with Pax6, Tbr2, and NeuroD2 antibodies or with an EdU staining kit at E16.5. CP, cortical plate; IZ, intermediate zone; SVZ, subventricular zone; VZ, ventricular zone. The scale bar is 50 μm. (B) Quantification of GFP+ cells co-expressing the markers. n ≥ 3 independent biological repeats. The results are expressed as the mean ± SD, and comparisons were performed by ANOVA. The statistically significant P values are shown as ∗P < 0.05, ∗∗P < 0.01 or ∗∗∗P < 0.001. [file Image_5.TIF]
