# Supplementary material for: Long Non-coding RNA T-uc.189 Modulates Neural Progenitor Cell Fate by Regulating Srsf3 During Mouse Cerebral Cortex Development
Source: Front Neurosci. 2021 Jul 20;15:709684. doi: 10.3389/fnins.2021.709684 (PMC8329457; doi:10.3389/fnins.2021.709684)
Supplement: Supplementary file 6 [file Table_1.DOCX]

**TABLE S1** The protein-coding potential of full-length T-uc.189.

| RNA | CPAT | CPC |
| --- | --- | --- |
| T-uc.189(full-length) | 0.45446856779665 | 1.54499 |
| Hes5(mRNA) | 0.94424764305393 | 3.27995 |
| Malat1(lncRNA) | 0.05599899518947 | -0.275281 |

**TABLE S2** Primers and oligonucleotide sequences for this study.

| Name | Sequences information |
| --- | --- |
| T-uc.189-ISH-F | CCCAAGCTTGTACTTGAGAGAGCACCTTTTAGAG |
| T-uc.189-ISH-R | CGCGGATCCGGTGGTGAGAAGAGACATGATGG |
| Srsf3-ISH-F | CCGGAATTCACAGTTGAAATGGAGGCATGC |
| Srsf3-ISH-R | CGCGGATCCACAAGGCACTAAGCCAGTGG |
| T-uc.189-NB-F | CCCAAGCTTGCAGCTGTTTTAATGTTACAACTGG |
| T-uc.189-NB-R | CGCGGATCCGGATTCAAGAACGGATGATTGGG |
| T-uc.189-RT-F | CCTTCGCCAACCAACTAAATCC |
| T-uc.189-RT-R | CCAGCAGTAAACTGTATAAGCAGG |
| Srsf3-RT-F | GCGCAGATCCCCAAGAAGG |
| Srsf3-RT-R | ATCGGCTACGAGACCTAGAGA |
| Gapdh-RT-RF | CTGCACCACCAACTGCTTAG |
| Gapdh-RT-RR | GTGGATGCAGGGATGATGTTC |
| T-uc.189-5’RACE | GGTTGGCGAAGGGGGTGTTGTGG |
| T-uc.189-3’RACE | CCCAGGCTGGCCAGTCGTCAGGTTGC |
| T-uc.189-shRNA-1 | GCTAGTAGGAGCAGGTATTTC |
| T-uc.189-shRNA-2 | GGTAGTTAGTAACTTCTATCT |
| T-uc.189-shRNA-3 | GGTGATAGGGAACTCATAATG |
| Srsf3-shRNA- 1 | GCTCCAGTATGGTAGGTATAT |
| Srsf3-shRNA- 2 | GGTGTTTGCTAGAGGTCATTA |
| Srsf3-shRNA- 3 | GCTTAGTGCCTTGTCTTAACT |
| shNC | GCGCGATAGCGCTAATAATTT |
